# The Selection of Sports Schools: The Influence of the Environment on the Development of Youth Athletes' Career Adaptability

**DOI:** 10.1111/sms.14767

**Published:** 2024-12-12

**Authors:** Jaakko A. O. Nikander, Asko Tolvanen, Tatiana V. Ryba

**Affiliations:** ^1^ Department of Psychology University of Jyväskylä Jyväskylä Finland

**Keywords:** career adaptability, development environments, dual career, youth athletes

## Abstract

There are many routes to achieving an elite status in sports, which can be described as a journey that involves many developmental environments. In terms of navigating transitions and across environments, the adolescent years are particularly crucial, and psychosocial skills may assist youth athletes to maintain balance and wellbeing in combining sports and education. The aim of this study was to investigate the environmental differences in career adaptability profiles as students adapt to their sports high schools. A total of 391 student‐athletes (51% female, 49% male) from six sports high schools in Finland participated in the study. To compare the environments' features, we created a typology of the environments based on a document analysis methodology. Our quantitative analysis builds upon the findings of the first author, who applied latent profile analysis (LPA) to career adaptability measures across the first year of sports high school. To investigate whether student‐athletes have a higher probability of belonging to a profile in certain sports high schools, we used multinomial regression analysis. The results showed that the most sports‐focused and centralized environment did not support career adaptability development to the same extent as other environments. Furthermore, we found that student‐athletes in the “highly successful” environment had the highest probability of belonging to the high or increased career adaptability profile, despite reporting the highest number of sports training hours and high academic success. This study demonstrates the importance of the locality and the authenticity of the environments and of how their features support student‐athletes' career adaptability development.

## 1 Introduction

After numerous years of experience in the field of talent development, Henriksen and Stambulova [1] stated that successful athletic development results from positive environments that support the holistic development of athletes. In terms of navigating such environments, the adolescent years are particularly crucial, not only because of the increasing demands of sports and intensified competition, but also because of the overlapping period when important developmental milestones are typically achieved. During the adolescent years, individuals are expected to explore future career options and to expand their resources to prepare and adapt to several transitions in the pursuit of their desired path [2]. When constructing a career, young people select available and compatible environments and integrate activities into their lives based on their characteristics, interests, competencies, and needs [3]. One potential career aim that may be pursued during adolescence is an elite sports career. Traditionally, sports‐related career paths have provided a clear structure through elements such as age‐specific performance expectations and talent development pathways [4]. The development of athletic talent begins at an early age, and more structures and institutionalization have been constructed to facilitate young athletes' career progression [4] suggesting that elite sports may be becoming more systematic than ever before, incorporating more normative pathways.

As environments can serve as resources or barriers for the development and well‐being of athletes, Henriksen and Stambulova [1] have highlighted the need for career support programs to focus on helping athletes prepare for and manage transitions between career stages. Therefore, for the holistic development of young athletes, career construction and related skills may be particularly important [5, 6]. Furthermore, it is necessary to investigate how environments differ in their capacity and functions to provide appropriate support to promote adolescent student‐athletes' dual career development. The aim of this study was to investigate the environmental differences in holistic development (i.e., career adaptability profiles) by creating an understating of the factors that may distinguish environments within the same national dual career system.

### 1.1 Developmental Environments

In the last decade, the development of athletic talent has been examined from a holistic and social perspective [1, 7, 8, 9]. Holistic development means that development has multiple dimensions (athletic, psychosocial, psychological, academic, athletic); changes in one dimension, such as transition to sport school, lead to changes in other areas of life, such as psychological, social and educational areas [7]. Since adolescents are responsible for both their education and for preparing for their future beyond the domain of sports, dual careers (i.e., integration of sport with education or work) have been proposed as a pivotal means to promote sustainable career development (e.g., the fulfillment of developmental tasks) and the growth of talented athletes [10, 11]. To integrate the different areas of young athletes' lives and facilitate a balanced life, dual career development environments (DCDEs) are recommended for young athletes as purposefully developed systems that combine athletic and educational domains [11, 12]. The holistic ecological approach has shifted attention from an individual focus to an environmental one [8, 13, 14, 15]. However, a recent study conducted by Thompson et al. [16] demonstrated the complex nature of managing youth athletes' holistic development. They highlighted the need to examine individual differences on an environmental level and to investigate how they may impact student‐athletes' trajectories, as the holistic impact may vary considerably depending upon, for example, athletes' gender.

The ECO‐DC project [9] mapped the field of ecological talent development by analyzing and comparing several environments across Europe and identified 10 success‐related characteristics of DCDEs [17], which can be roughly classified into philosophical (e.g., empowering approach, mental health support, holistic development) and structural characteristics (e.g., flexible studies, support team, expert services). As the holistic ecological approach (HEA) is in its infancy, Henriksen and Stambulova [1, p. 9] invited researchers to contribute to HEA‐informed research to “create environments that facilitate athletes' successful pursuit of career excellence.” However, various challenges involved in evaluating environments arise from the complexity of such environments, as they encompass multiple interrelated settings, levels, and domains, as well as histories. Since the environmental evaluation literature has emphasized a case study approach, few studies have compared environments with similar structural, functional, and philosophical systems (i.e., nationally built and developed systems).

HEA‐informed case studies have also been examined through a critical lens. Feddersen et al. [18] problematized the duality of previous environmental evaluations, which were regarded as either successful or unsuccessful, with a strong emphasis on the successful ones. Furthermore, Feddersen et al. [18] have noted that previous studies have not accounted for the ambiguity of the environment, positing that environments may compensate for some dysfunctional practices or a lack of resources by implementing well‐functioning practices. In addition, most studies have been “real‐time” studies, leaving a gap in investigations of the developmental process. In their mixed method systematic review, Thompson et al. [19] focused on athletes' developmental processes by examining the impact of sports schools on holistic development, highlighting the ambiguity of the functions of the environment. They uncovered various immediate short‐ and long‐term positive and negative impacts associated with the athletic, academic, psychological, and psychosocial development of student athletes. For example, positives included enhanced athletic development, more stable levels of well‐being, and augmented qualities and skills applicable to other areas of life. Negatives included school absenteeism, lower tertiary attainment, limited experiences of ordinary life outside competitive sports, reduced agency for alternative careers, and challenges finding new directions in life (e.g., highlighting the importance of career adaptability, for example). Furthermore, authors found that there was also a high incidence of injury, dropping out of school, loss of family time, and pressure from parents to perform. They concluded that practitioners can therefore promote or detract from positive outcomes by designing an appropriate development environment. The authors also highlighted that the wide range of data collection methods used to evaluate the impact of sport school programs makes it difficult to compare the results of different studies.

### 1.2 Career Construction

Career construction theory posits a transactional relationship between athletes and the environments in which such athletes are embedded [3]. This means that an individual's career‐related goals and perceived competencies guide their behavior, and these experiences operate in both directions, providing feedback that influences educational and career trajectories [3]. In their athletic career development path, athletes may face ambiguous and complex challenges related to developmental tasks (i.e., exploring different roles), transitions (i.e., between schools and clubs), and traumas (i.e., de‐selection, dropping out) that the athlete must resolve to preclude negative development or to be able to pursue their goals [3]. In addition, young athletes must confront the social world around them and the assumptions around development. For example, parents' guidance and assumptions about career decisions may differ significantly from coaches' views about prioritizing sports, which can intensify ambiguity among athletes [20]. Therefore, in order for adolescent student‐athletes to navigate their life journeys in different environments, they should be supported in their career construction behaviors and resources.

### 1.3 Career Adaptability

To navigate different environments and achieve positive development, Savickas [3] suggests that career adaptability is crucial: “Individuals can adapt more effectively if they face changing conditions with increasing awareness and information seeking, followed by decision making, trial behaviours leading to stable commitment projected forward for a period of time and active role management, and forward‐looking disengagement” [3, p. 156]. Career adaptability can be regarded as a form of self‐regulatory competence. The word “adapt” means the following: “bringing inner needs and outer possibilities into harmony, indicating success, satisfaction and well‐being” [3, p. 156]. This suggests that the environment must also provide support to foster this harmony. Career adaptability as a psychosocial skill enables young people to regulate their career strategies along four dimensions (i.e., concern, control, curiosity, and confidence). Concern refers to the individual's level of the awareness and preparedness for near and distant career development tasks and transitions. Career control refers to an individual's efforts to take responsibility for their career and to choose how to approach career development tasks. Career curiosity refers to the behavior of gathering information, exploring new experiences and roles, and reflecting on the alignment between the individual and a particular career. Career confidence refers to the self‐efficacy to pursue a self‐determined career and to cope successfully with difficulties. In addition, Ryba et al. [21] introduced a dimension of dual career concern in athletic contexts, which refers to an individual's awareness of the challenges of combining athletic and academic goals and being prepared for their DC path.

Keeping talented youth athletes in sports requires that they can successfully adapt to transitions and maintain their wellbeing. Individuals with high career adaptability possess the readiness and resources to adapt to shifting contexts. In previous studies examining student‐athletes, Nikander et al. [22], found that throughout high school, dual career adaptability remained stable, and individual variations explained differences in adaptabilities rather than time‐specific variations, indicating that the development of career adaptability was not facilitated by the sports schools. Ojala et al. [23] investigated career adaptability profiles across sports high schools. They found that lower career adaptability profiles of student‐athletes were overrepresented among those who withdrew from their athletic career (i.e., competitive sports); such students also reported lower GPAs (i.e., grade point average), suggesting that career adaptability influences student‐athletes' career development. It has been shown that students who perform well in school (e.g., with a high GPA) may demonstrate higher orientation and preparation for future careers [24]. It becomes also known that some youth athletes opt out of athletic career by the end of high school [11]. When investigating environmental influences, it becomes evident that parents' expectations regarding their children's success predict career adaptability; the more strongly the parents believe in their child's success, the stronger their children's career adaptability levels are [25]. Subsequently, Gamboa et al. [26] suggested that socio‐emotional skills are important for fostering career behaviors by acting as a mediator between parental support and career exploration.

In navigating the environments, student‐athletes may need to move away from their homes to access the dual career development environments. Although the transition may promote the development of career adaptabilities (Savickas, Timonen et al.) [3, 27], a high sport focus may also have a negative impact on overall development. Korhonen et al. [20] suggested that a sport‐focused culture and environment where the individuals are surrounded by other like‐minded individuals may not encourage exploration of future career possibilities or provide opportunities for growth. Savickas [3], for example, suggests that personal development is brought about by change, which is triggered by other people. Furthermore, lack of balance between different domains in DCDEs can lead to stress and insufficient support for interests outside of sport, eventually leading to the dropout from sport [28]. In addition, the boarding school environment can be highly structured, controlling and protective, as shown by Storm et al. [17] in a study of essential features in DCDEs across Europe. The heavy training and study loads, as well as the predetermined schedules, limited the student‐athletes' interaction with peers outside the context of elite sport context in a Belgian elite sports school.

Thompson et al. [19] highlighted that the holistic impact of the sports school may vary considerably depending on the gender of the athlete. For example, their study showed that females had lower sport‐confidence. Ryba et al. [29] argued that dual careers are assembled through gendered social processes, with young female athletes often displaying less motivation toward athletic careers [30] as a result of navigating the intersecting pressures of societal expectations and competing commitments in education and sport. According to career construction theory, involvement in different career‐related activities is culturally saturated: [3] society places different expectations on young men and women, for example, women's limited professional career opportunities in sports may lead to expectations to pursue dual careers [29], while men may face attitudes that they should aim for a professional sports career. In football in 2017 a report by the International Federation of Professional Footballers showed that elite sport is not a real career path in itself for the majority of the players [31]. The gender gap has also been demonstrated in the career adaptability of student‐athletes [22]. In the Nikander et al.'s [22] study, adolescent women athletes demonstrated lower career adaptability across sports high schools supporting the findings that DCDEs may be more compatible for males.

### 1.4 The Present Study

To provide resources for athletes' developmental journeys, career adaptability could be an important resource. In addition to focusing on the longitudinal development of student‐athletes' career adaptability, we can gain more information about the career developmental processes of adolescent athletes. Understanding the features that contribute to functional and dysfunctional developmental environments may provide crucial information to support sports school programs. In the present study, we aimed to develop an understanding of how sports high schools differ in terms of the development profiles corresponding to their student‐athletes' career adaptability and, subsequently, what environmental characteristics may be associated with these differences.

The present study has examined the following research questions:

1. How do sports high schools differ in terms of their student‐athletes' career adaptability profiles?
2. What environmental features may be related to the differences in the student‐athletes' career adaptabilities?

Since previous studies have revealed that gender [24, 25], education [23, 32], and sports achievements [24, 33] are related to career adaptability, we included these in the analysis.

## 2 Methodology

Our research builds upon the previous findings from Nikander et al.'s [14, 25] studies examining student‐athletes' holistic development. Specifically, our analysis builds upon the findings of Nikander et al. [25], which examined what kinds of distinct career adaptability profiles could be identified among youth athletes at the transition stage to a sports high school. The findings of these studies prompted us to investigate how different environments support student‐athletes' holistic development differently.

### 2.1 Participants and Procedures

The present study is part of the Longitudinal Dual Career Study [34], which followed adolescent athletes' experiences at sports high schools. A total of 391 student‐athletes (51% female, 49% male) from six sports high schools in Finland participated in the study. The mean age of the student‐athletes when the first measurement was performed was 16 years (SD = 0.17). Half of the student‐athletes participated in individual sports (e.g., athletics, biathlon) and half in team sports (e.g., football, ice‐hockey). Student‐athletes represented their sports from regional level to the international level and had been competing in their sports for an average of 7 years (SD = 2.41). Additionally, a typology of the environments was created by combining the Finnish Longitudinal Dual Career Study data set with follow‐up data from the environments (collected annually from the years 2016/2017). The follow‐up data collection was conducted by the Research Institute for High Performance Sports (KIHU).

To compare the environments in their attempts to support holistic development, we selected national high‐performance environments (i.e., elite sports high schools) that deploy similar structures, systems, and resources to support athletes' dual careers. Sports high schools are one of the identified DC pathways [12] in Finland. In this study student‐athletes enrolled in six sports high schools (two each from the Northern, Central, and Southern parts of Finland). These sports high schools prioritized different sports; for example, at some, the emphasis is on the area having facilities for winter sports. Hence, student‐athletes' mobility may force adolescents to move out from their homes to pursue their athletic dreams and to find a compatible environment. Sports high schools have certain sports that are supported more than others. For example, smaller sports (based on the number of athletes in that particular sport) may not have specific support for coaching but otherwise provide the same benefits to athletes.

Ethical approval was obtained from the ethics committee of the respective university (in Finland, ethical approval is generally sought from university ethics committees). The participants signed informed consent forms before participating in the study. In Finland, informed consent from the parents or guardians of young people over 15 is not required. The data were collected at the schools using an online questionnaire or an identical paper questionnaire. The data were collected at two different time points: T1 (autumn of the first year in sports high school) and T2 (spring of the first year in sports grammar school). Career adaptability was assessed at each time point (T1‐T2) using self‐report scales.

### 2.2 Environments

To understand the differences between the environments, we attempted to construct a typology of the environments based on document analysis. Categorization was conducted by the first author and the third author, who was involved in a process of reviewing the first author's assumptions. A deductive coding approach was used, and information from the combined data was transferred into pre‐defined categories inspired by the DCDE taxonomy of Morris et al. [12] Categories included the location, level of centralization, number of sports, gender distribution, support for studies, and education and sports emphasis.

### 2.3 Measurements

Dual‐career adaptability was measured using the Career Adapt‐Abilities Scale‐Dual Career form (CAAS‐DC) [21, 35]. The CAAS‐DC contains a total of 27 items measuring five dimensions of career adaptability: concern (four items; e.g., planning how to achieve my goals), control (six items; e.g., taking responsibility for my actions), curiosity (six items; e.g., investigating options before making a choice), confidence (six items; e.g., working up to my ability), and dual career concerns (five items; e.g., becoming aware of the sport choices that I must make). Previous research with Finnish sports high school students has shown that the CAAS‐DC has factorial and concurrent validity [21]. All items were scored on a five‐point Likert scale (1 = not one of my strongest skills; 5 = one of my strongest abilities). A mean variable was created for each subscale, indicating competence in that dimension. The Cronbach's alphas for the scores of the different subscales at the two time points (T1 and T2) varied between 0.82 and 0.91.

### 2.4 Statistical Analysis

**FIGURE 1:**
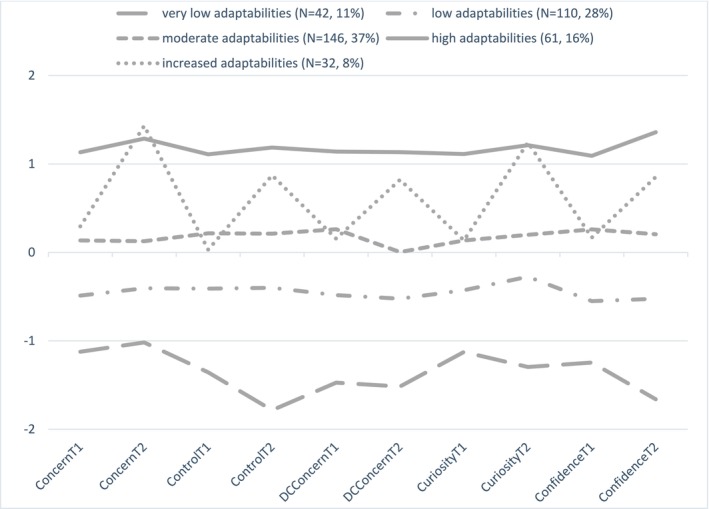
Identified career adaptability profiles among student‐athletes across the first grade of sports high school in Nikander et al.'s [25] study. T1 = Time 1 (the Fall of the first grade); T2 = Time 2 (the Spring of the first grade).

Our analysis builds upon the findings of Nikander et al. [25], which applied a latent profile analysis (LPA) to career adaptability measurements from two time points and found five latent classes as the best fitting model for the data (*N* = 391). The profiles are presented in the Figure 1. These latent classes were labeled as follows: (1) stable very low adaptability (scores of −1 SD below the mean), (2) stable low adaptability (scores below the mean), (3) stable moderate adaptability (scores above +1 SD the mean), (4) stable high adaptability (scores above +1 SD the mean), and (5) increased adaptability (+1 SD above the means at T2). To find statistically significant factors that predicted members of the latent classes, we used multinomial regression analysis R3STEP [36] implemented in Mplus version 8.8 [37]. This method produced estimates using each latent class as a reference group, allowing all possible pairwise comparisons. Even the initial LPA used all available data full information maximum likelihood, in the R3STEP drops cases having missing values in predictors (in our analysis, the number of dropped cases was six). We used preliminary test for each of the variable using DCAT and BCH tests implemented in Mplus to get the main effect of each variable in the model. Main effect of school $\left(\chi^{2}\left(16\right)=48.31,p<0.001\right)$, gender $\left(\chi^{2}\left(4\right)=10.29,p=0.036\right)$, and sports achievements $\left(\chi^{2}\left(16\right)=43.92,p<0.001\right)$ was statistically significant. Correspondingly main effect of GPA was not statistically significant $\left(\chi^{2}\left(4\right)=7.33,p=0.119\right)$. In the multinomial logistic regression using R3STEP analysis, we selected all four environment variables even the preliminary result for GPA was not statistically significant. The model consists of 32 parameters whose statistical significance for Type I error could be corrected using Bonferroni correction (observed *p* = 0.001 corresponds Bonferroni corrected *p* < 0.001). This correction justified including the GPA in the model. The results consist of estimates, standard errors, p‐values, odds‐ratios, and 95% confidence intervals for odds‐ratios. As descriptive information, we found expected odds based on initial LPA without predictors.

## 3 Results

### 3.1 Typology of the Environments

Based on the typology, we categorized the six sports high schools studied in the Finnish Longitudinal Dual Career Study as follows: A = education‐focused, B = specialized, C = sports‐focused, D = balanced, E = highly successful (in sport and academic), or F = sports‐friendly environments. The following are the descriptive summaries of the environments. Table 1 presents the background information regarding the environments.

**TABLE 1:** Descriptive information regarding the participants in the environments.

School	*N*	# of sports	% of student‐athletes	G.P.A. (SD)	Gender (women/men)	Location	Sports level (% of top 3 in their sports)	TimeS (SD)	TimeE (SD)	Living (% of moved)
A	29	7	16	8.9 (0.55)	62/38	Rural	10	22.1 (8.9)	14.5 (15.4)	34.5
B	50	6	48	8.7 (0.63)	40/60	Rural	34	22.6 (9.6)	7.9 (7.8)	64
C	136	24	70	8.9 (0.68)	60/40	Urban	49	25.9 (8.6)	8.2 (7.3)	4.4
D	62	13	28	8.8 (0.47)	50/50	Urban	39	25.5 (9.4)	12.2 (8.3)	9.7
E	69	14	41	9.0 (0.58)	46/54	Urban	44	27.0 (7.5)	8.7 (10.3)	7.2
F	45	11	13	8.6 (0.76)	38/62	Urban	33	23.7 (10.1)	10.8 (11.1)	11.1

*Note: N* = number of participants in the environment, # number of sports = number of priority sports, % of student‐athletes = percentage of student‐athletes in the environment, G.P.A. = grade point average on a scale from 4 to 10, gender = percentage of adolescent men and women, sports level = what percentage of student‐athletes have placed top 3 in national championships in their sports, TimeS = estimated time devoted to sports in a week, TimeE = estimated time spent studying outside of school in a week, living = percentage who has moved out of their home.

“A” represents a rural environment where almost a third of the student‐athletes have moved away from home. It is limited to certain sports. Athletes are a minority in the environment compared to the number of students in the environment (i.e., 1/7). Compared to other environments, their sporting success is the lowest, but students in this environment spend the most time on education, making it an “education focused” environment. Student‐athletes have the potential to receive 47 h of study counseling per year. In the first year, student‐athletes make a plan for their high school trajectory. Parents are also involved in executing their plan. Sports facilities are located nearby, making it a time‐efficient environment.

“B” is a “specialized” type of environment where most of the student‐athletes have moved away from their homes making this environment also a specialized environment. Half of the students at the high school are athletes. Such a school also specializes in a small number of sports. It has a relatively high success rate in relation to the number of sports. In addition, student‐athletes from this environment have achieved high success in the senior ranks as well. Student‐athletes in this environment spend less time on education compared to other environments. Resources for study counseling include 19 h of support per student‐athlete in a year. For the first year, student‐athletes will receive a predetermined schedule, based upon which they devise their own plan. The plan can be modified based on the competition schedules and training camps (e.g., national team camps or international championships). Coaches are included in the counseling processes. The environment is compact, as athletes live near the training locations.

“C” is a “sports focused” and centralized environment with all the services nearby. Two thirds of the students in the high school are athletes. It has the highest number of priority sports and the highest success rate in sports. It cooperates closely with the sports clubs. In addition, athletes who graduate from this environment have the highest success rates based on the senior rankings. Study counseling is arranged in a manner wherein study plans are devised at the beginning of high school, and they have a periodic follow‐up system. Study counselors responsible for athletes follow the students' educational progress; however, they may have many students in their group.

“D” is a “balanced environment” in terms of the time devoted to sports and education and success in education and sports. One fourth of the students are athletes in this environment. Some of the graduated athletes have achieved success in senior championships. It engages in local cooperation with different stakeholders in the field of education and sports. It is an urban environment where training facilities are located around the city. From the study support recourse perspective, there is total of four study counselors for all the athletes.

“E” is a “highly successful” environment. Student‐athletes in this environment have high success rates both in education and in sports. It supports multiple sports, and student‐athletes in this environment devote the most time to sports. A bit less than half of the students in this environment are athletes. It engages in close cooperation with the city, not only in terms of sports but also education, which makes it unique compared to other environments. Coaches and study counselors have close cooperations and weekly meetings where they consider dual‐career and individual student‐athletes' needs. They also offer ongoing individual study counseling services. Many sports facilities and training places are near the school.

“F” is a “sports friendly” environment where some of the students move away from their parents. It offers sports friendly support, as it collaborates with the Research Institute for High Performance Sports and the Faculty of Sports Science. All the training facilities are nearby. In addition, it cooperates with the respective city's sports bureau. Less than 15% of the students are athletes in this environment. Student‐athletes have an annual study counseling interview, and they are supported and recommended to take sports into consideration when planning their studies. In addition, counseling is focused on recognizing skills that are learned from sports that can be transferred to other domains of life. Student‐athletes who have graduated from this environment have also achieved success in senior rankings.

### 3.2 Career Adaptability Profiles in the Sports High Schools

The aim of the study was to examine whether student‐athletes have a higher probability of belonging to a certain profile in a certain sports high school. First, in Table 2 cross‐tabulation of the career adaptability profiles is presented (χ^2^ (20, *N* = 391) = 45.33, *p* = 0.001) showing that environments differ in their career adaptability profiles. The expected odds for the environments are shown in Table 3. In addition, in Table S1 all the expected odds (including the non‐significant ones) are presented. The results showed that there were differences in the probability of a student‐athlete from a particular sports high school belonging to a particular profile. In particular, student‐athletes from the most sports focused environments had higher odds to belong to the lower career adaptability profiles compared to the highly successful and balanced environments. Students in education‐focused environments had a four times higher probability of belonging to the stable moderate adaptability profile than to the stable very low profile compared to students in sports‐focused environments. Students in balanced environments had a nearly five times higher probability of belonging to the stable high adaptability profile than to the stable very low profile compared to students in sports‐focused environments. In addition, students in high‐success environments had an over six times higher probability of belonging to the stable high adaptability profile than the stable very low profile compared to students in sports‐focused environments. This difference between very low adaptability profiles and high adaptability profiles was also explained by gender and grade point average. Adolescent men had a two times higher probability of belonging to a high adaptability profile than to the stable very low profile; having a higher GPA doubled the probability of belonging to the high adaptability profile compared to the stable very low profile. When comparing the stable very low adaptability profile to the increased adaptability profile, we found that students in specialized environments had over seven times, and those in highly successful environments had a 14 times higher probability of belonging to the increased adaptability profile than to the stable very low adaptability profile compared to students in sports‐focused environments. Furthermore, students in education‐focused environments had four times, and students in a highly successful environment had a nearly four times higher probability of belonging to a high adaptability profile than a stable high adaptability profile relative to students in sports‐focused environments. In addition, belonging to a high adaptability profile was explained by gender, GPA, and sports levels; being an adolescent men, having a higher GPA, and being at a higher level in sports increased the probability of belonging to the high adaptability profile than the stable high adaptability profile. Similar results were found when comparing the stable moderate adaptability profile and stable high adaptability profile. Finally, when comparing the stable low adaptability profile and increased adaptability profile, students in specialized environments and high‐success environments had an over seven times higher probability of belonging to an increased adaptability profile than a stable low adaptability profile in sports‐focused environments. Similar differences were found when comparing moderate profiles to increased profiles in high‐success environments.

**TABLE 2:** A descriptive cross‐tabulation of the career adaptability profiles.

School	Profile					
	C1	C2	C3	C4	C5	Total
A						
Count	8	7	7	6	1	29
% with school	27.6%	24.1%	24.1%	20.7%	3.4%	100%
Adj. residuals	−1.1	−0.5	2.4	0.8	−1.0	
B						
Count	17	14	6	6	7	50
% with school	34.0%	28.0%	12.0%	12.0%	14.0%	100%
Adj. residuals	−0.5	0.0	0.3	−0.8	1.6	
C						
Count	67	40	11	14	4	136
% with school	49.3%	29.4%	8.1%	10.3%	2.9%	100%
Adj. residuals	3.6	0.4	−1.2	−2.1	−2.8	
D						
Count	20	20	8	12	2	62
% with school	32.3%	32.3%	12.9%	19.4%	3.2%	100%
Adj. residuals	−0.9	0.8	0.6	0.9	−1.6	
E						
Count	17	16	6	16	14	69
% with school	24.6%	23.2%	8.7%	23.2%	20.3%	100%
Adj. residuals	−2.4	−1.0	−0.6	1.9	4.0	
F						
Count	17	13	4	7	4	45
% with school	37.8%	28.9%	8.9%	15.6%	8.9%	100%
Adj. residuals	0.1	0.1	−0.4	0.0	0.2	100%

**TABLE 3:** Expected odds based on an initial LPA.

Profile	School	Estimate	S.E.	*p*	Odds‐ratio	95% CI
C1 vs. C3	A	1.422	0.676	0.035	4.145	1.10–15.59
C1 vs. C4	D	1.559	0.608	0.10	4.753	1.43–15.66
	E	1.882	0.586	0.001	6.569	2.01–20.73
Gender		0.840	0.406	0.038	2.317	1.05–5.10
G.P.A		0.941	0.336	0.005	2.561	1.33–4.95
C1 vs. C5	B	1.988	0.789	0.012	7.302	1.56–34.30
	E	2.694	0.704	0.000	14.787	3.72–58.77
C2 vs. C4	A	1.459	0.732	0.046	4.30	1.03–18.05
	E	1.333	0.572	0.020	3.791	1.24–11.63
Gender		0.820	0.403	0.042	2.271	1.03–5.00
G.P.A		1.112	0.338	0.001	3.039	1.57–5.89
Level		0.356	0.170	0.036	1.428	1.02–1.99
C2 vs. C5	B	2.003	0.826	0.015	7.413	1.47–37.39
	E	2.144	0.700	0.002	8.533	2.16–33.64
C3 vs. C4						
Gender		1.221	0.491	0.013	3.390	1.30–8.87
G.P.A		1.012	0.413	0.014	2.754	1.26–6.19
Level		0.381	0.180	0.034	1.464	1.03–2.08
C3 vs. C5	E	1.90	0.84	0.023	6.685	1.30–34.39

*Note:* Odds for the environments: (A) education‐focused 0.21; sports‐friendly (F) 0.33; balanced (D) 0.45; specialized (B) 0.36; highly successful (E) 0.51. Reference sports‐focused (C). C1 = stable very low adaptability, C2 = stable low adaptability, C3 = stable moderate adaptability, C4 = stable high adaptability, C5 = increased adaptability.

## 4 Discussion

In the present study, we aimed to develop an understanding of how sports high schools differ in terms of the development profiles corresponding to their student‐athletes' career adaptability and, subsequently, what environmental characteristics may be associated with these differences. In particular, we focused on the transition process during the first year of sports high school. We found differences in the probability of a student‐athlete from a particular sports high school belonging to a particular profile, suggesting that environmental characteristics have an impact on the development of career adaptability in student‐athletes. Specifically, it appears that the most sport‐focused and centralized environments do not support career adaptability development to the same extent as other environments. On the other hand, we found that student‐athletes in the “highly successful” environment had the highest probability of belonging to the high or increased career adaptability profile, despite reporting the highest amount of training hours in sports. Students in this environment also exhibited high academic success in addition to sporting success, suggesting that a well‐functioning dual career development environment supports young people's sporting ambitions, educational aspirations, and psychosocial skills and career development.

In this study, students in highly successful, education focused and balanced environments had a higher probability of belonging to the higher career adaptability development profiles compared to the sports‐focused environment. This finding is important as career adaptabilities has been shown to be associated with the well‐being [22] and career outcomes [23] of adolescent athletes, the selection of sport school may be crucial to their development. It appears that rural environments foster higher career adaptabilities compared to the most sports‐focused and centralized environments. One explanation could be that adolescents in the rural environments have moved out of their homes, resulting in greater levels of responsibility, adaptations to new situations, as well as future orientations, which has been shown to increase career adaptability [3, 38, 39]. Although student‐athletes in all the environments were in a transitioning phase, that is a phase when adaptability becomes evident [27, 40]; it may be that the pre‐determined and normative pathways of sports high schools [11, 14] inhibit this development. For example, in this study, sports‐focused environments had the most structured systems by providing predetermined study plans. Additionally, although the “specialized” environment was characterized as having a highly‐predetermined structure and strong emphasis on sports, it may be that changing one's life situation and taking more responsibility for one's life is necessary to develop adaptability skills [3].

The results of this study suggest that reduced sports prioritization, as was evident in the education‐focused and balanced environments, may be a source of career adaptability development in sports high schools. As career adaptability is psychosocial in its nature and individuals construct their careers in interaction with experiences and people in their environment, it may be that student‐athletes in environments that have a majority of non‐athlete students experience superior career adaptability development. For example, numerous studies have shown that athletes' social relationships are closely linked to athletic domain sports [20, 41, 42]. Due to the presence of more non‐athlete students in the environments and in the classes, student‐athletes in these environments have the potential to experience different social situations and to collect versatile career stories and plans. In addition to peer‐student influences, nearby adults who serve as coaches can also influence student‐athletes' future perspectives and career adaptability [26, 43]. The environments with less prioritized sports also had higher coach‐athlete ratios, which may contribute to closer relationships with the coaches and provide socio‐emotional support, which may be a source of career adaptability as well [26].

A distinctive feature distinguishing the environments was support for study. For example, highly successful environments fostered close cooperation and weekly meetings discussing dual career and individual student‐athletes' needs. Furthermore, in this environment, student‐athletes could receive ongoing study‐counseling. Taking into consideration that the information is shared between education and sports domains, study counselors may be more efficient, as they know their student‐athletes' situations. Being familiar with the student‐athletes' situations may facilitate conversations and the relevant questions to enhance career‐related thinking [3]. In addition, in education‐focused environments, during the first year, student‐athletes devise a plan for high school, whereas in other environments, the plan is made for the first year. This kind of future orientation (i.e., career concern and curiosity) may facilitate career‐related thinking. Special features of this environment was that parents were also involved in executing the plan. As parents have been proven to influence student‐athletes' career adaptability [25, 26], involving parents in the planning may be a factor in determining why student‐athletes in this environment had a higher probability of belonging to the higher career adaptability profiles compared to the sports‐focused environments.

It appears that being an adolescent man is correlated with belonging to a higher career adaptability profile. As we were measuring dual career adaptability, it became evident that adolescent men may have higher career prospects in sports compared to adolescent women, which suggests that men may be more confident in their future possibilities. For example, Thompson et al. [7] showed that adolescent women had lower sports confidence compared to adolescent men in the sports schools. This may also create contradictory messages for adolescent women: They should prioritize sports but lack real opportunities for professional careers in sports. This message may complicate youth athletes' career construction. Overall, Thompson et al. [7] highlighted that holistic impacts may be gender‐dependent, and further support maybe be required for female student‐athletes in sports school environments. As adolescent women have been shown to have challenges with balancing a dual career, especially in the transition phase to sports high school [7, 44], promoting psychosocial skills (e.g., enhanced confidence) and empowering adolescent women in the DCDEs is important, as they may demonstrate lower adaptive career behaviors. This also raises critical questions about how sports development environments are embedded in structures that prioritize male athletes and their needs, potentially obstructing the holistic development and career transitions of female athletes.

Considering that the students in all the investigated environments are high achievers based on their GPA and sports success, it is interesting that we found distinct developmental trajectories environmentally. This highlights the importance of developing the environments holistically and accounting for certain features of the functional environments. For the environments to develop their functions, it is important to consider whether they incorporate well‐functioning features and aim to improve their dysfunctional features, as suggested by Henriksen and Stambulova [1]. Although the structural dual career systems were similar in the studied environments, it seems that the dual career support processes vary between the environments: Some environments better support their student‐athletes' career development than others. This highlights the need for locality‐based and bottom‐up solutions in the developmental process; for example, highly successful environments had close cooperations with stakeholders, not only in sports but also in educational institutions; conversely, sports‐focused environments mostly had cooperation with sports clubs. Regarding the sustainability of the environments, it should be more transparent how the environments function so that youth athletes can receive the same kind of support independent of the environment, and when choosing their environments, they should be able to trust that their development and growth are equally supported.

This study indicates that it is important to study environments in parallel to be able to recognize features that support student‐athletes' career adaptability. Therefore, we recommend that sports high schools have a follow up system and evaluations of the most important features. The strength and novelty of the study is that it compares multiple environments. It also demonstrates the importance of the locality and authenticity of the environments and how their features support student‐athletes' career adaptability. The findings illustrate that it is crucial to recognize socio‐cultural aspects and how the environment has been developed and to account for these factors when developing the environments in parallel to top‐down policies. Therefore, it is important that environments recognize the functions that support career development and to acknowledge how they can develop their environment in a way that supports student‐athletes' career development. For example, this study suggests that student‐athletes in sports‐focused environments demonstrated lower odds of belonging to higher career adaptability profiles compared to other environments, indicating that high sports focus may not be the optimal solution to promote youth athletes' career development. In Finland, it is also important for sports high schools to share their best practices to develop well‐functioning features, especially considering that sports environments are coordinated and developed by the national Olympic Committee according to their elite sports strategies.

The present study has some limitations that should be taken into account before generalizing the results. Although we had an extensive qualitative data to conduct a typology of the environments, we did not have the opportunity to conduct observational or interview data of the environments according to the HEA, which could have given us a richer description of the environments. Furthermore, there was a large variation in the living percentage who has moved out of their home, thus, it would be important to examine what kind of career adaptability profiles student‐athletes not living home would demonstrate. Future research should focus on the developmental processes of the environments longitudinally, as well as the interventions to support student‐athletes' psychosocial resources (i.e., career adaptabilities) to navigate across the environments. In addition, it should be important for the stakeholders in the sports schools to think in advance about the demands that student‐athletes will face in their future careers. Future research would also benefit from knowing how student‐athletes in different sports differ in their development of career adaptability, for example, whether there are differences in sports that offer greater opportunities for professional status.

## 5 Perspectives

The present study contributes to the existing literature in three ways. First, we found differences in the probability of a student‐athlete from a particular sports high school belonging to a particular profile, and environmental characteristics have an impact on the development of career adaptability in student‐athletes. As career adaptability has been shown to be associated with the well‐being and career outcomes of adolescent athletes, the selection of sport school may be crucial to their development. Second, the most sport‐focused and centralized environments did not support career adaptability development to the same extent as other environments. At the same time, we found that student‐athletes in the “highly successful” environment had the highest probability of belonging to the high career adaptability profile, despite reporting the highest number of sports training hours and high academic success, suggesting that high sport focus alone may not be the most optimal solution for supporting adolescent athletes' career preparation. Third, the findings illustrate that it is crucial to recognize socio‐cultural aspects (e.g., patriarchal structures) and how the environment has been developed in alignment with top‐down policies.

## Conflicts of Interest

The authors declare no conflicts of interest.

## Supporting information


Table S1.

## Data Availability

The data that support the findings of this study are available from the corresponding author upon reasonable request.
