# Supplementary material for: The Selection of Sports Schools: The Influence of the Environment on the Development of Youth Athletes' Career Adaptability
Source: Scand J Med Sci Sports. 2024 Dec 12;34(12):e14767. doi: 10.1111/sms.14767 (PMC11635908; doi:10.1111/sms.14767)
Supplement: Supplementary file 1 — Table S1. [file SMS-34-e14767-s001.docx]

Table S1. Expected odds based on an initial LPA analysis

| Profile | School | Estimate | S.E. | P-value | Odds-ratio | 95% CI |
| --- | --- | --- | --- | --- | --- | --- |
| C1 vs. C2  Gender  G.P.A  Level | A  F  D  B  E | 0.271  0.036  0.630  -0.015  0.550  0.020  -0.171  -0.191 | 0.695  0.512  0.465  0.546  0.506  0.334  0.286  0.134 | 0.69  0.94  0.18  0.98  0.28  0.95  0.55  0.15 | 1.311  1.036  1.878  0.985  1.733  1.020  0.843  0.826 | 0.34-5.12  0.38-2.83  0.76-4.70  0.34-2.87  0.64-4.70  0.53-1.96  0.48-1.48  0.64-1.07 |
| C1 vs. C3  Gender  G.P.A  Level | A  F  D  B  E | 1.422  0.370  1.108  0.623  0.794  -0.380  -0.072  -0.215 | 0.676  0.655  0.584  0.660  0.656  0.419  0.346  0.135 | .035  0.57  0.058  0.346  0.226  0.364  0.835  0.111 | 4.145  1.448  3.029  1.864  2.212  0.684  0.931  0.806 | 1.10-15.59  0.40-5.23  0.96-9.52  0.51-6.80  0.61-8.01  0.30-1.56  0.47-1.83  0.62-1.05 |
| C1 vs. C4  Gender  G.P.A  Level | A  F  D  B  E | 1.730  1.138  1.559  1.125  1.882  0.840  0.941  0.166 | 0.771  0.694  0.608  0.678  0.586  0.406  0.336  0.158 | 0.025  0.10  0.01  0.10  .001  .038  .005  0.29 | 5.639  3.122  4.753  3.079  6.569  2.317  2.561  1.180 | 1.24-25.58  0.80-12.59  1.43-15.66  0.82-11.62  2.01-20.73  1.05-5.10  1.33-4.95  0.87-1.61 |
| C1 vs. C5  Gender  G.P.A  Level | A  F  D  B  E | 0.705  1.090  0.635  1.988  2.694  0.827  0.463  -0.064 | 1.365  0.919  0.998  0.789  0.704  0.483  0.447  0.139 | 0.61  0.24  0.52  .012  .000  0.10  0.30  0.64 | 2.025  2.974  1.888  7.302  14.787  2.286  1.589  0.938 | 0.14-29.40  0.49-18.02  0.27-13.34  1.56-34.30  3.72-58.77  0.89-5.90  0.66-3.82  0.72-1.23 |
| C2 vs. C3  Gender  G.P.A  Level | A  F  D  B  E | 1.151  0.335  0.478  0.638  0.244  -0.400  0.099  -0.025 | 0.719  0.710  0.590  0.717  0.682  0.439  0.378  0.150 | 0.11  0.64  0.42  0.37  0.72  0.36  0.79  0.87 | 3.162  1.398  1.613  1.893  1.276  0.670  1.104  0.976 | 0.77-12.95  0.35-5.62  0.51-5.13  0.46-7.72  0.34-4.86  0.28-1.58  0.53-2.32  0.73-1.31 |
| C2 vs. C4  Gender  G.P.A  Level | A  F  D  B  E | 1.459  1.103  0.929  1.140  1.333  0.820  1.112  0.356 | 0.732  0.715  0.613  0.719  0.572  0.403  0.338  0.170 | .046  0.12  0.13  0.11  .020  .042  .001  .036 | 4.300  3.013  2.531  3.126  3.791  2.271  3.039  1.428 | 1.03-18.05  0.74-12.24  0.76-8.42  0.76-12.79  1.24-11.63  1.03-5.00  1.57-5.89  1.02-1.99 |
| C2 vs. C5  Gender  G.P.A  Level | A  F  D  B  E | 0.435  1.054  0.005  2.003  2.144  0.807  0.635  0.127 | 1.343  0.945  1.006  0.826  0.700  0.484  0.447  0.145 | 0.75  0.27  0.97  .015  .002  0.10  0.16  0.38 | 1.544  2.870  1.005  7.413  8.533  2.241  1.886  1.135 | 0.11-21.48  0.45-18.29  0.14-7.21  1.47-37.39  2.16-33.64  0.87-5.79  0.79-4.53  0.85-1.51 |
| C3 vs. C4  Gender  G.P.A  Level | A  F  D  B  E | 0.308  0.768  0.451  0.502  1.088  1.221  1.012  0.381 | 0.813  0.832  0.715  0.844  0.732  0.491  0.413  0.180 | 0.71  0.36  0.53  0.55  0.14  .013  .014  .034 | 1.360  2.155  1.569  1.652  2.970  3.390  2.754  1.464 | 0.28-6.70  0.42-11.01  0.38-6.37  0.32-8.65  0.71-12.48  1.30-8.87  1.26-6.19  1.03-2.08 |
| C3 vs. C5  Gender  G.P.A  Level | A  F  D  B  E | -0.717  0.719  -0.473  1.365  1.90  1.207  0.535  0.152 | 1.387  1.042  1.073  0.934  0.84  0.564  0.508  0.160 | 0.61  0.49  0.66  0.14  .023  0.03  0.29  0.34 | 0.488  2.053  0.623  3.917  6.685  3.344  1.708  1.164 | 0.03-7.41  0.28-15.82  0.08-5.11  0.63-24.10  1.30-34.39  0.87-5.89  0.66-3.82  0.72-1.23 |
| C4 vs. C5  Gender  G.P.A  Level | A  F  D  B  E | -1.024  -0.048  -0.923  0.863  0.811  -0.013  -0.477  -0.229 | 1.401  1.064  1.062  0.945  0.754  0.521  0.476  0.180 | 0.47  0.96  0.39  0.36  0.28  0.98  0.32  0.23 | 0.359  0.953  0.397  2.371  2.251  0.987  0.621  0.795 | 0.02-5.59  0.12-7.67  0.05-3.18  0.37-15.12  0.51-9.87  0.36-2.74  0.24-1.58  0.59-1.13 |

Odds for the environments: (A) Education-focused .21; Sports-friendly (F) .33; Balanced (D) .45; Specialized (B) .36; Highly successful (E) .51. Reference Sports-focused (C). C1 = stable very low adaptability, C2 = stable low adaptability, C3 = stable moderate adaptability, C4 = stable high adaptability, C5 = increased adaptability.
